# Pseudophosphorylation of *Arabidopsis* jasmonate biosynthesis enzyme lipoxygenase 2 *via* mutation of Ser^600^ inhibits enzyme activity

**DOI:** 10.1016/j.jbc.2023.102898

**Published:** 2023-01-10

**Authors:** Diljot Kaur, Sonia Dorion, Souleimen Jmii, Laurent Cappadocia, Jacqueline C. Bede, Jean Rivoal

**Affiliations:** 1 Department of Plant Science, McGill University, Quebec, Canada; 2 Institut de Recherche en Biologie Végétale, Université de Montréal, Montréal, Quebec, Canada; 3 Département de Chimie, Université du Québec à Montréal, Montréal, Quebec, Canada

**Keywords:** enzyme kinetics, lipoxygenase pathway, phosphorylation, enzyme structure, site-directed mutagenesis, 13*S*-lipoxygenase 2, protein structure prediction, jasmonate, AA, arachidonic acid, AtLOX2, arabidopsis lipoxygenase 2, JA, jasmonic acid, JA-Ile, (+)-7- iso-jasmonoyl-isoleucine, JAZ, Jasmonate-Zim, LA, linoleic acid, LOX, lipoxygenase, MS, mass spectrometry, MS/MS, tandem MS, OPDA, 12-oxo-phytodienoic acid, PUFA, polyunsaturated fatty acid, α-LeA, α-linolenic acid

## Abstract

Jasmonates are oxylipin phytohormones critical for plant resistance against necrotrophic pathogens and chewing herbivores. An early step in their biosynthesis is catalyzed by non-heme iron lipoxygenases (LOX; EC 1.13.11.12). In *Arabidopsis thaliana*, phosphorylation of Ser^600^ of AtLOX2 was previously reported, but whether phosphorylation regulates AtLOX2 activity is unclear. Here, we characterize the kinetic properties of recombinant WT AtLOX2 (AtLOX2^WT^). AtLOX2^WT^ displays positive cooperativity with α-linolenic acid (α-LeA, jasmonate precursor), linoleic acid (LA), and arachidonic acid (AA) as substrates. Enzyme velocity with endogenous substrates α-LeA and LA increased with pH. For α-LeA, this increase was accompanied by a decrease in substrate affinity at alkaline pH; thus, the catalytic efficiency for α-LeA was not affected over the pH range tested. Analysis of Ser^600^ phosphovariants demonstrated that pseudophosphorylation inhibits enzyme activity. AtLOX2 activity was not detected in phosphomimics Atlox2^S600D^ and Atlox2^S600M^ when α-LeA or AA were used as substrates. In contrast, phosphonull mutant Atlox2^S600A^ exhibited strong activity with all three substrates, α-LeA, LA, and AA. Structural comparison between the AtLOX2 AlphaFold model and a complex between 8R-LOX and a 20C polyunsaturated fatty acid suggests a close proximity between AtLOX2 Ser^600^ and the carboxylic acid head group of the polyunsaturated fatty acid. This analysis indicates that Ser^600^ is located at a critical position within the AtLOX2 structure and highlights how Ser^600^ phosphorylation could affect AtLOX2 catalytic activity. Overall, we propose that AtLOX2 Ser^600^ phosphorylation represents a key mechanism for the regulation of AtLOX2 activity and, thus, the jasmonate biosynthesis pathway and plant resistance.

Oxylipin jasmonate phytohormones coordinate key processes in plant growth and development and are necessary for plant resistance against necrotrophic pathogens and chewing herbivore pests, such as beetles and caterpillars (1, 2, 3, 4). During the foliar response to these biotic stresses, dynamic and rapid changes lead to increased levels of jasmonate phytohormones and robust plant defense responses. In particular, the extremely rapid surge (<30 s) in jasmonate levels after stress initiation suggests that posttranslational modification of constitutive jasmonate biosynthetic enzymes may be an important mechanism for the regulation of this pathway (5, 6, 7).

Jasmonates, such as 12-*oxo*-phytodienoic acid (OPDA), jasmonic acid (JA), and (+)-7- *iso*-jasmonoyl-isoleucine (JA-Ile), are derived from 18C galactolipids of the chloroplast envelope and thylakoid membrane (1, 8). In response to wounding, fatty acid desaturases and galactolipases catalyze the production of α-linolenic acid (α-LeA, C18:3) from lipids making up these chloroplastic membranes (9). Then, a key step of jasmonate biosynthesis takes place with the oxygenation of α-LeA by a 13*S*-lipoxygenase (LOX, EC 1.13.11.12) (10). The resulting (13*S*)-hydroperoxyoctadecatrienoic acid is converted to the bioactive intermediate (9*S,*13*S*)-OPDA through the action of allene oxide synthase and allene oxide cyclase (11, 12). After transfer from the plastid to the peroxisome (13, 14), OPDA is converted to JA via a reduction followed by three β-oxidations (15). JA is transported into the cytoplasm where it is converted to its bioactive form, JA-Ile, by conjugation with isoleucine catalyzed by Jasmonate Resistant1 (JAR1) (16). JA-Ile acts as a bridge to bring together transcriptional repressor Jasmonate-Zim domain (JAZ) proteins and COI1, a F-box protein associated with the SCF^COI1^ complex (17). Ubiquitination and 26*S*-proteasome–mediated degradation of JAZ transcription regulators releases MYC transcription factors from repression, resulting in jasmonate-responsive gene expression (18, 19). In *Arabidopsis thaliana* (arabidopsis), jasmonate-responsive gene expression includes transcription factors and enzymes involved in defensive specialized metabolite pathways, such as glucosinolate biosynthesis, and genes that encode enzymes in jasmonate biosynthesis. This results in a robust feedforward activation of the jasmonate pathway, key for a strong defense response. However, this pathway is also modulated by negative regulation, which involves transcriptional and posttranslational regulation (8, 20, 21, 22). Alternatively, the (13*S*)-hydroperoxyoctadecatrienoic acid generated through LOX activity may be converted into green leaf volatiles that play an important role within plant defense signaling, through the hydroperoxide lyase pathway (23, 24, 25).

The 13*S*-LOX catalyzing an early step in the pathway is a possible key regulator for metabolic flux into oxylipin biosynthesis. This enzyme belongs to a class of iron-containing dioxygenases that catalyze hydroperoxidation reactions by adding molecular oxygen to the pentadiene moiety (5 carbon chain with two double bonds surrounding a methylene group in the middle (−CH=CH−CH_2_−CH=CH−) of polyunsaturated fatty acid (PUFA) substrates, such as α-LeA or linoleic acid (LA) (26). Depending on the site of oxygenation of the fatty acid, plant LOXs are distinguished as 9*S*- or 13*S*-LOX. In arabidopsis, there are six members in the LOX gene family (27). Two of these (*AtLOX1* and *AtLOX5*) are 9*S*-LOXs and four (*AtLOX2*, *AtLOX3*, *AtLOX4*, and *AtLOX6*) are 13*S*-LOXs involved in jasmonate biosynthesis, suggesting some functional redundancy. In foliar-wounded plants, AtLOX2 is highly correlated with the local jasmonate burst, whereas AtLOX6 is associated with the vasculature and thus, thought to be primarily responsible for the production of jasmonates or related compounds that move through the plant and activate defense responses, including *AtLOX2, AtLOX3*, and *AtLOX4* expression (28, 29), thereby contributing to the systemic jasmonate increases. AtLOX3 and AtLOX4 are also involved in the regulation of the wound-associated attenuation of growth (30).

The jasmonate burst in response to foliar damage is initiated less than a minute after the stress, suggesting that constitutive enzymes involved in jasmonate biosynthesis are likely posttranslationally regulated, allowing the plant to respond in a prompt and dynamic fashion (5, 6, 7). In a proteomic study, the constitutive phosphorylation of AtLOX2 at the Ser^600^ was identified (31). In contrast, this enzyme was dephosphorylated in wounded arabidopsis leaves, suggesting that AtLOX2 activity may be regulated by reversible phosphorylation of Ser^600^. These data suggest that AtLOX2 could be less active when phosphorylated, although phosphorylation may also influence other aspects of protein function (32).

The present work tests the hypothesis that phosphorylation of AtLOX2 on Ser^600^ inhibits enzyme activity. To address this question, we first characterized enzyme kinetic studies of recombinant WT AtLOX2 (AtLOX2^WT^) using three substrates, α-LeA, LA, or arachidonic acid (AA), over a pH range (7.0–8.8). In arabidopsis leaves, α-LeA (18:3) and LA (18:2) are endogenous substrates of LOX2 (33) and α-LeA serves as precursor for the jasmonate biosynthetic pathway (8). The enzyme catalyzes the hydroperoxidation of these substrates generating a precursor that can give rise to various stress-associated metabolites including C6 volatile compounds and traumatin, as well as the important defense signaling molecules OPDA or JA-Ile. AA (20:4), on the other hand, is rarely found in seed plants and not considered a natural substrate for plant LOXs (34). It is nevertheless used for *in vitro* characterization of plant LOXs (27, 35, 36). AA is abundant in plant oomycete pathogens, such as *Phytophthora* species, and released into plant tissues during the infection process as well as believed to activate the jasmonate-responsive pathway (34, 37).

We then compared the AtLOX2^WT^ enzyme kinetics to the phosphomimic (Atlox2^S600D^). We additionally analyzed a phosphonull variant (Atlox2^S600A^) and a mutant where Ser^600^ was changed to Met (Atlox2^S600M^) to approximate the bulkiness of the phosphoSer side chain without the negative charge. Kinetics of the phosphovariants using α-LeA, LA, and AA as substrates were tested at pH 7.0 and 8.2, respectively, representing two extreme values of chloroplast stromal pH in the dark or in the light. These analyses were complemented by protein modeling which support the view that phosphorylation of Ser^600^ affects AtLOX2 catalytic activity.

## Results

### Production and purification of soluble AtLOX2^WT^ and its variants Atlox2^S600A^, Atlox2^S600D^, and Atlox2^S600M^

Recombinant proteins lacking the chloroplast transit peptide were produced in *Escherichia coli* HB101l- (Fig. S1*A*). Conditions for producing soluble AtLOX2^WT^ and variant (Atlox2^S600A^, Atlox2^S600D^, Atlox2^S600M^) proteins were optimized with respect to culture parameters such as temperature and length of induction. Optimal production was achieved by overnight induction at 17 °C in the presence of 0.6 mM IPTG (Fig. S1*A*). His-tagged AtLOX2^WT^ and variants were purified over a Ni-NTA column (Figs. S1*B* and S2). The purified recombinant proteins migrated on a 10% (w/v) SDS-PAGE at a size of ∼98.7 kDa, the expected size of the recombinant protein. A minor contaminant appeared just below the 98.7 kDa band. These two bands were recognized by a commercially available polyclonal antibody previously used to detect AtLOX2 on immunoblots (38) (Fig. S2*B*), suggesting that they both correspond to AtLOX2 polypeptides.

To confirm that the purified AtLOX2^WT^ was not phosphorylated on Ser^600^ by a promiscuous bacterial protein kinase, a sample of the recombinant protein was analyzed by LC–tandem-mass spectrometry (MS) (MS/MS). This generated 1332 total spectra, providing a coverage of 97% for the protein. There was no phosphorylation detected on Ser, Thr, or Tyr residues on the entire protein. A total of 12 spectra corresponding to tryptic peptides containing Ser^600^ were recovered (Fig. S3). As evidence for an absence of phosphorylation on Ser^600^, the fragmentation spectra and ion tables for all the peptides carrying Ser^600^ recovered after LC-MS/MS sequencing are shown (Fig. S3). Therefore, kinetic data (below) were obtained with an unphosphorylated AtLOX2 recombinant enzyme.

### AtLOX2 activity and substrate saturation kinetics

**Figure 1:**
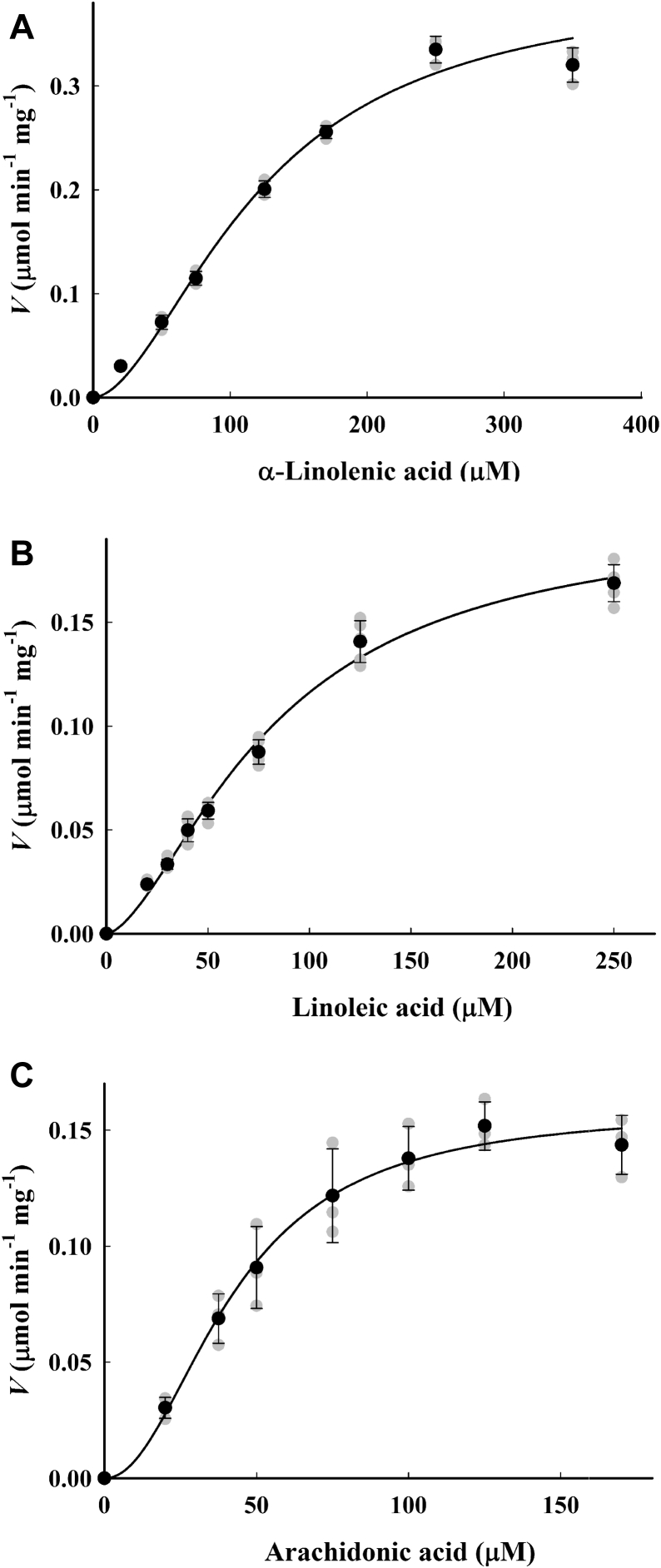
**AtLOX2 substrate saturation kinetics showing cooperative behavior.** Recombinant AtLOX2^WT^ was assayed with (*A*), α-linolenic acid; (*B*), linoleic acid; or (*C*), arachidonic acid at pH 8.2. The line is the fit to the sigmoidal curve generated by the Hill equation using SigmaPlot 12.5. *Black* symbols and error bars represent the mean ± SD. Individual temporal replicates (n = 3–5) appear as *gray* symbols. AtLOX2, arabidopsis lipoxygenase 2.

Substrate saturation kinetics studies were carried out with three different substrates, α-LeA, LA, and AA. Physiological pH values of the chloroplast stroma ranging from 7.0 to 8.2 (meant to reflect contrasting chloroplast stroma pH values during dark and light conditions, respectively) were used, as well as more alkaline pH values (8.5 and 8.8). Activity measurements were taken under steady state conditions, and the short lag period previously observed with a soybean enzyme (39) was not detected. For all substrates, positive cooperative binding was observed as denoted by Hill constant values >1 (Fig. 1 and Table 1). For α-LeA and LA, this effect was generally more pronounced at neutral than alkaline pH values.

**Table 1:** Recombinant AtLOX2^WT^ kinetics

Substrate	Parameter (mean ± SD)	pH					
		**7.0**	**7.5**	**7.9**	**8.2**	**8.5**	**8.8**
α-Linolenic acid	*V*_*max*_ (μmol min^-1^ mg^-1^)	0.17 ± 0.03^2,b^	0.23 ± 0.04^2,a^	0.23 ± 0.01^2,b^	0.42 ± 0.08^1,a^	0.42 ± 0.05^1,a^	0.45 ± 0.08^1,a^
	*S*_0**.5**_ (μM)	61.3 ± 5.8^2,a^	91.6 ± 20.4^1,2,a^	111.7 ± 48.0^1,2,a^	108.3 ± 17.2^1,2,a^	168.9 ± 30.5^1,a^	154.9 ± 46.9^1,a^
	*k*_cat_ (s^-1^)	0.28 ± 0.04^2,b^	0.38 ± 0.07^2,a^	0.38 ± 0.02^2,b^	0.70 ± 0.13^1,a^	0.70 ± 0.08^1,a^	0.75 ± 0.13^1,a^
	*k*_cat_/*S*_0.5_ (s^-1^ mM^-1^)	4.62 ± 0.37^1,b^	4.19 ± 0.18^1,b^	4.07 ± 2.31^1,a^	5.58 ± 0.19^1,a^	4.19 ± 0.35^1,a,b^	5.10 ± 1.36^1,a^
	*h*	2.7	1.9	1.7	1.9	1.3	1.6
Linoleic acid	*V*_*max*_ (μmol min^-1^ mg^-1^)	0.07 ± 0.01^3,c^	0.11 ± 0.03^2,3,b^	0.09 ± 0.02^3,c^	0.20 ± 0.01^1,b^	0.15 ± 0.03^2,b^	0.13 ± 0.02^2,b^
	*S*_0.5_ (μM)	26.7 ± 2.5^3,c^	35.8 ± 3.2^3.b^	38.4 ± 6.7^3,b^	82.6 ± 6.3^1,b^	68.5 ± 15.3^1,2,b^	62.4 ± 5.2^2,b^
	*k*_cat_ (s^-1^)	0.12 ± 0.02^4,c^	0.19 ± 0.05^3,4,b^	0.15 ± 0.03^3,4,c^	0.33 ± 0.02^1,b^	0.27 ± 0.07^1,2,b^	0.22 ± 0.03^2,3,b^
	*k*_cat_/*S*_0.5_ (s^-1^ mM^-1^)	4.56 ± 0.70^1,b^	4.56 ± 0.27^1,2,b^	3.83 ± 0.70^1,2,3,a^	4.07 ± 0.27^1,2,3,b^	3.54 ± 0.38^2,3,b^	3.43 ± 0.42^3,b^
	*h*	3.4	2.1	1.6	1.6	1.8	1.7
Arachidonic acid	*V*_*max*_ (μmol min^-1^ mg^-1^)	0.22 ± 0.02^1,2,a^	0.26 ± 0.02^1,a^	0.29 ± 0.04^1,a^	0.15 ± 0.03^2,b^	0.17 ± 0.05^2,b^	0.15 ± 0.02^2,b^
	*S*_0.5_ (μM)	38.5 ± 1.8^2,b^	56.2 ± 2.7^1,2,b^	86.6 ± 18.5^1,a,b^	40.7 ± 7.2^2,c^	57.7 ± 17.4^1,2,b^	45.6 ± 10.6^2,b^
	*k*_cat_ (s^-1^)	0.37 ± 0.03^1,2,a^	0.44 ± 0.04^1,a^	0.49 ± 0.07^1,a^	0.24 ± 0.05^2,b^	0.29 ± 0.08^2,b^	0.24 ± 0.04^2,b^
	*k*_cat_/*S*_0.5_ (s^-1^ mM^-1^)	9.58 ± 0.71^1,a^	7.83 ± 0.53^1,a^	5.69 ± 0.49^2,a^	5.95 ± 1.00^2,a^	5.08 ± 0.52^2,a^	5.36 ± 0.35^2,a,b^
	*h*	2.1	1.8	1.4	2.1	1.9	2.2

Kinetic parameters were obtained across a range of pH values that covers the stromal conditions in the dark (pH 7.0) and the light (pH 8.2). Each value represents the mean ± SD (n = at least three temporal replicates). For a given parameter for each substrate, numbers indicate a significant difference at different pH values (2 factor ANOVA, *p* ≤ 0.05). Alphabetical lettering indicates significant differences between substrates for a given parameter. Statistical differences were not determined for the Hill coefficient (*h*).

**Figure 2:**
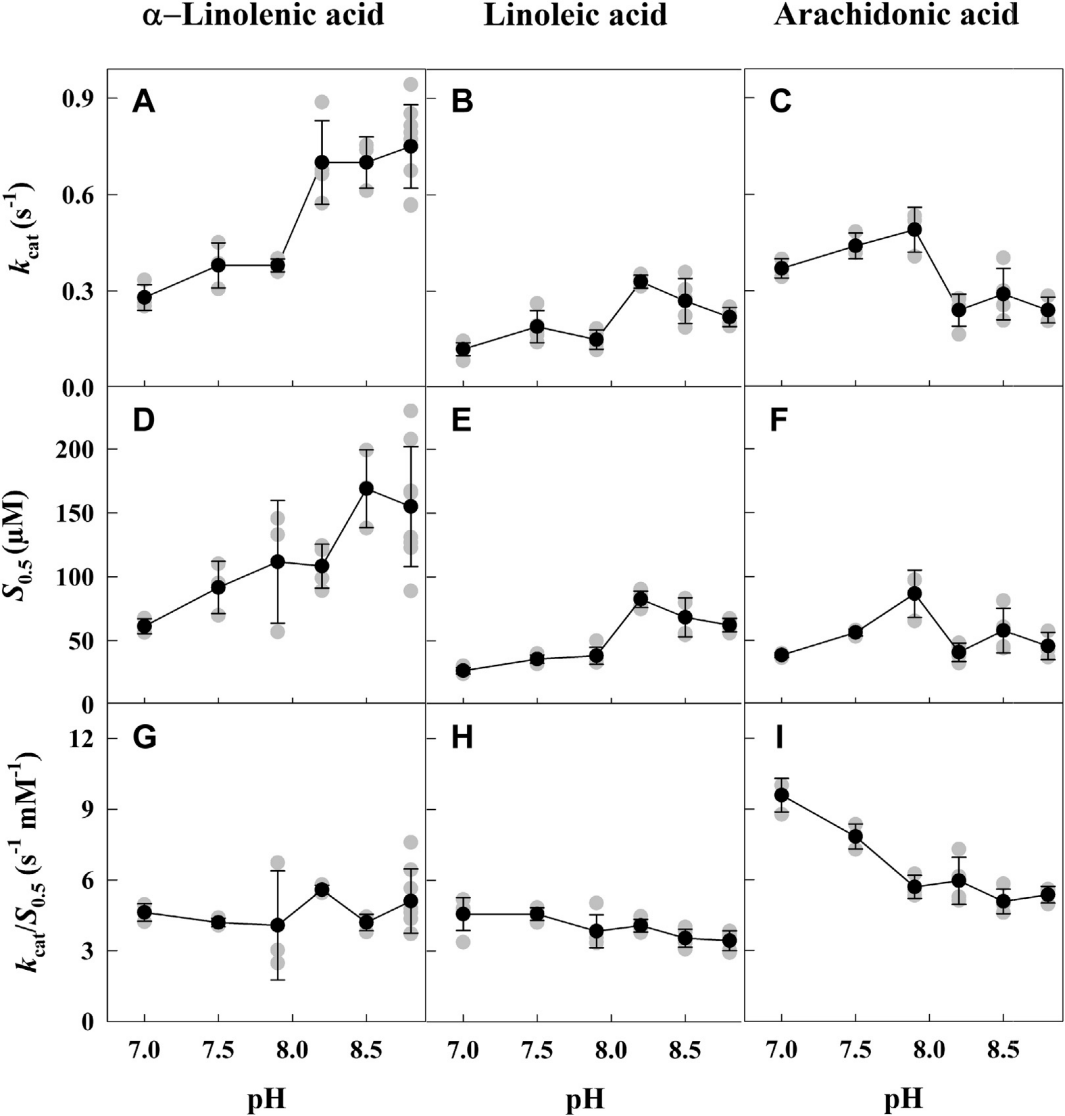
**Enzyme kinetics of recombinant AtLOX2**^**WT**^**.** Turnover number (*k*_cat_, A – C), substrate affinity (*S*_0.5_, D – F), and catalytic efficiency (*k*_cat_/*S*_0.5_, G – I) are shown for the three substrates, α-linolenic acid (*A*, *D* and *G*), linoleic acid (*B*, *E* and *H*), and arachidonic acid (*C*, *F* and *I*) over the pH range from 7.0 to 8.8. Significant differences between substrates and pH values are indicated in Table 1. *Black* symbols and error bars represent the mean ± SD. Individual temporal replicates (n = 3–8) appear as *gray* symbols.

The *V*_*max*_ and *k*_cat_ values of recombinant AtLOX2^WT^ for AA were highest under mildly alkaline pHs (7.5–7.9). In contrast, the *V*_*max*_ and *k*_cat_ for physiological substrates α-LeA and LA were higher under more alkaline conditions than neutral pH (Fig. 2 and Table 1). For α-LeA and LA, *V*_*max*_ values were respectively >2.4 and >2.8 times higher at pH 8.2 than pH 7.0. Specific activities obtained with α-LeA were also generally higher than those obtained with LA or AA. However, in comparison to AA, for which the substrate affinity was not affected by pH, the affinity of AtLOX2^WT^ for α-LeA and LA decreased importantly as the pH increased. The catalytic efficiency (*k*_cat_/*S*_0.5_) of AtLOX2^WT^ for α-LeA and LA did not change over the pH range reflecting a higher catalytic activity but lower substrate affinity at alkaline pH and the opposite at neutral pH. In contrast, AtLOX2^WT^ catalytic efficiency with AA as substrate generally decreased with more alkaline pH values.

### Conservation of the sequence containing the Ser^600^ phosphosite among plant 13S-LOXs involved in defense

**Figure 3:**
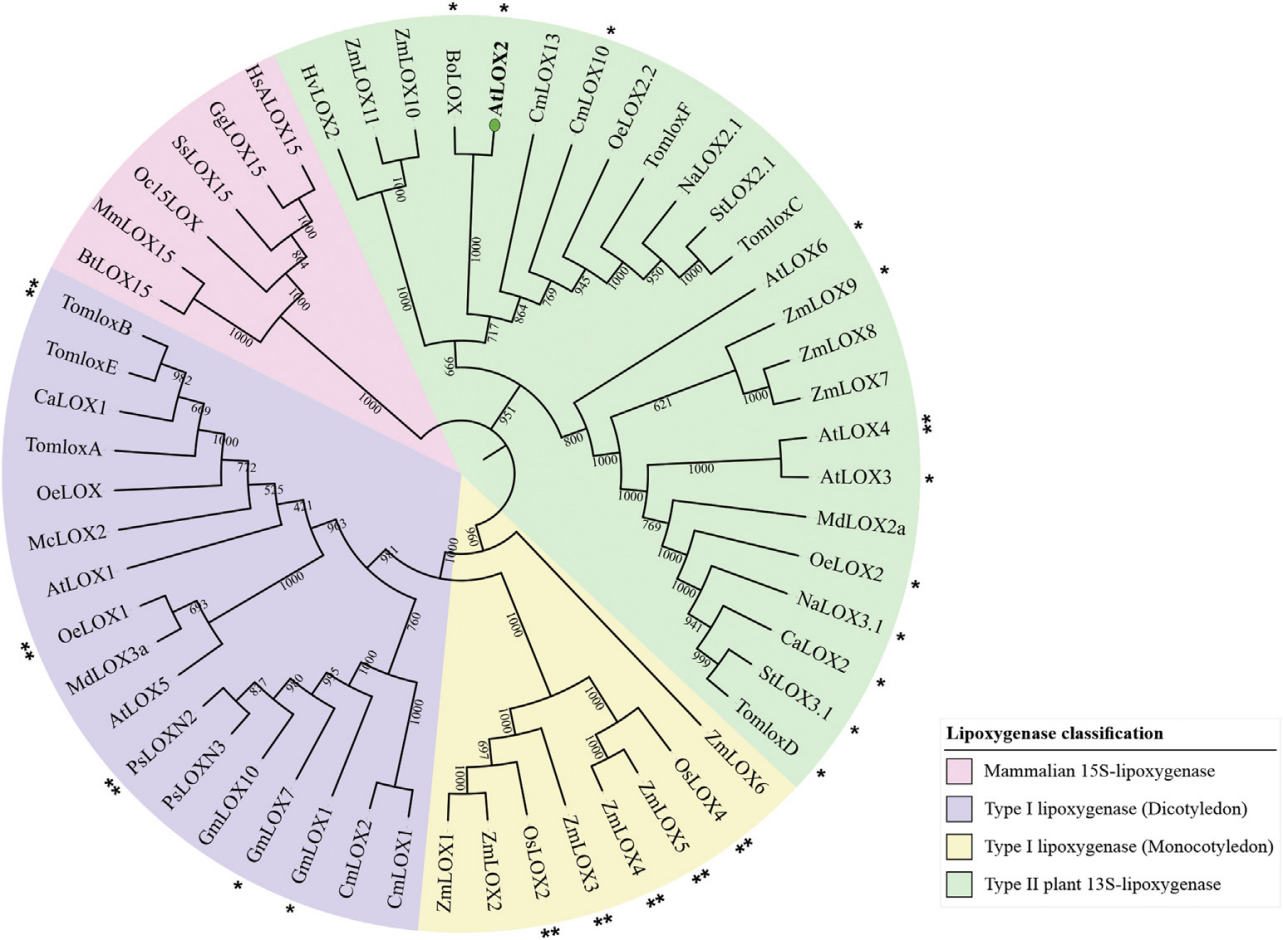
**Phylogenetic analysis and conservation of a putative Ser phosphorylation site in plant and animal lipoxygenases**. The phylogenetic relationship of 55 lipoxygenase amino acid sequences from organisms representing plants (monocots and dicots) and animals is shown. The full-length sequences of 55 LOX listed in Table S1 were used to generate the tree. A single asterisk “∗” represents lipoxygenases where the putatively phosphorylated Ser is present. A double asterisk “∗∗” represents sequences with a Thr instead of a Ser. The 21 amino acid sequences containing these residues in each protein are listed in Table S1. In AtLOX2, this residue is Ser^600^. LOX, lipoxygenase; AtLOX2, arabidopsis lipoxygenase 2.

AtLOX2 is constitutively phosphorylated at Ser^600^ in arabidopsis leaves and dephosphorylated in response to mechanical damage (31). Fifty-five protein LOX sequences of organisms spanning from plants to mammals were obtained from the NCBI database. The sequences were aligned to generate a phylogenetic tree (Fig. 3). The conservation of a Ser residue or a Thr residue at a position equivalent to that of AtLOX2 Ser^600^ was documented (Fig. 3 and Table S1). Based on sequence alignments, the Ser residue was not found in mammalian 15*S*-LOXs. In plants, 45% of these LOXs either maintained this Ser residue (27%) or had a Thr in its place (18%). The conserved Ser was mainly found in Type II LOXs, whereas a Thr was identified in its place in monocot Type I LOXs. Type II LOXs possess a chloroplast-targeting signal peptide on the N-terminus which is absent in Type I LOXs (40). Plant LOXs carrying the putative Ser phosphosite or Thr residues are highly associated with documented functions in plant defense or jasmonate synthesis (Table S1 and associated references therein). Of the 30 LOXs associated with plant defense, more than half of them possessed this putative Ser or Thr phosphosite. A Weblogo3 representation (41) of the 21 amino acid peptides surrounding this residue in the 30 LOXs shows that the only other prominent amino acid at this position is Ala (Fig. S4). Nevertheless, there are some exceptions, as not all plant LOXs associated with defense contain this putative phosphosite. Some enzymes, for example TomLoxB, OeLox1 and AtLOX4 or GmLOX1, contain a Thr or Ser, respectively, but have not been associated with plant defense so far; however, these examples are the minority.

### Ser^600^ is located on an arched helix bordering the substrate-binding pocket

**Figure 4:**
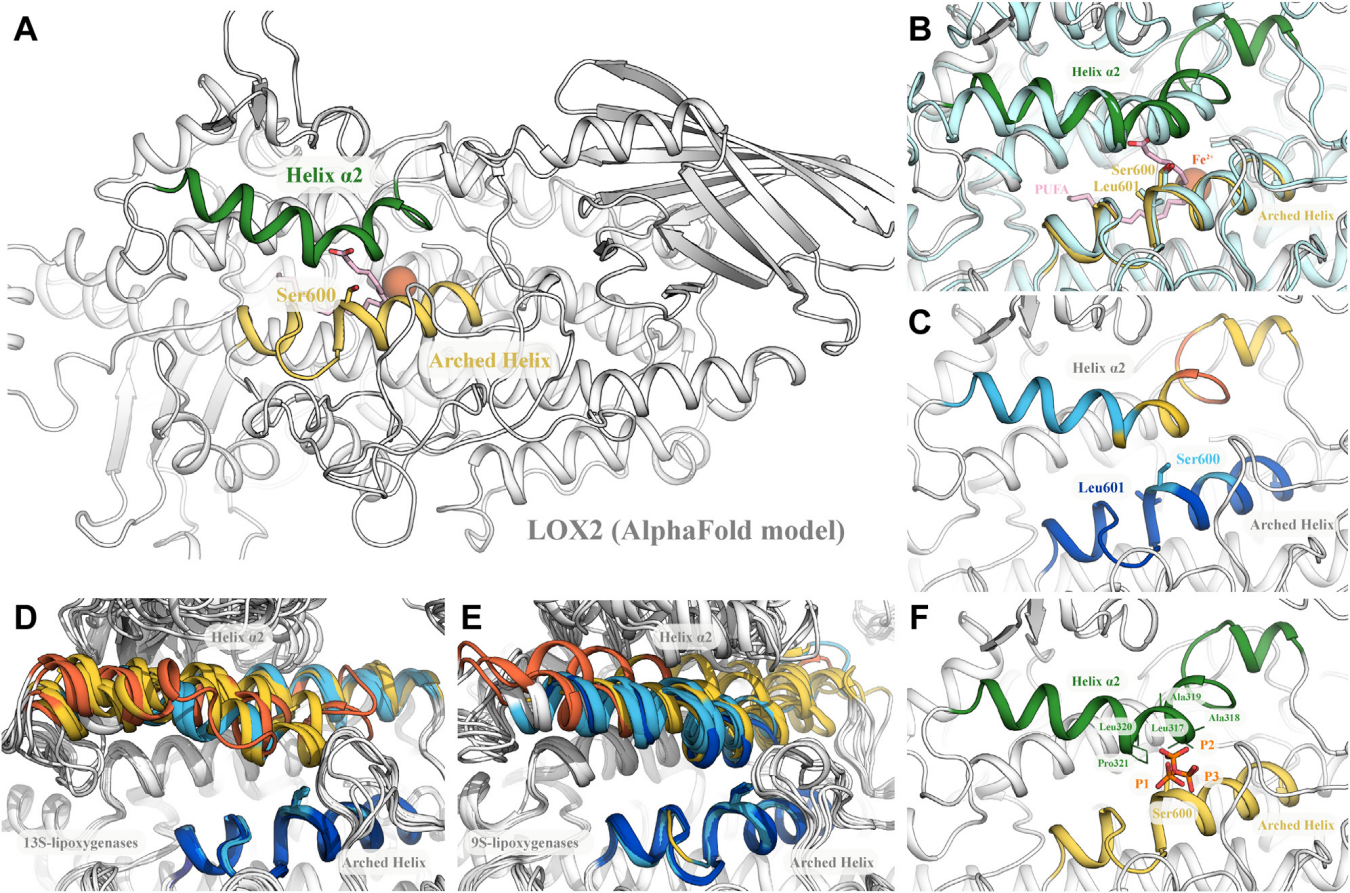
**Structural context of Ser**^**600**^**in the structure of LOX2 predicted by AlphaFold.***A*, global view of the structure of LOX2. Residues 1-70 that include a chloroplast transit peptide (amino acids 1–56) are predicted to be disordered and omitted for clarity. The arched helix encompassing Ser^600^ is colored in *yellow*, whereas helix α2 is colored in *green*. The position of two ligands, polyunsaturated fatty acid (PUFA) (in *pink*) and iron (in *orange*), were obtained from the lipoxygenase structures 4QWT and 1YGE after superimposition of their catalytic domain with LOX2. *B*, structural alignment between LOX2 and the crystal structure of soybean 13*S*-lipoxygenase 1 (1YGE; in *teal*). The position of the PUFA (in *pink*) corresponds to its relative position in the lipoxygenase structure 4QWT. *C*, representation of the pLDDT values of helix α2 and the arched helix of LOX2. Helix α2 and the arched helix are colored according to the pLDDT values: very high >90 (*blue*), confident >70 (*pale blue*), low>50 (*yellow*), very low <50 (*orange*). *D*, structural alignment between Type II 13-S lipoxygenases colored by pLDDT values. *E*, structural alignment between Type I 9-S lipoxygenases colored by pLDDT values. *F*, model of phosphorylated Ser^600^ of LOX2. Three different positions for the phosphate moiety are represented on Ser^600^ side chain oxygen atom. Side chains of residues clashing with phosphate are shown. LOX, lipoxygenase.

To understand the possible implications of Ser^600^ phosphorylation on LOX2 activity, we analyzed the structure of LOX2 predicted using AlphaFold (42, 43). In this model, the LOX2 catalytic domain (residues 202–896) adopts a canonical LOX fold (Fig. 4*A*) that is most similar to the structure of soybean 13*S*-LOX-1 as assessed by structural comparison using Dali and a RMSD of 2.9 Ǻ between LOX2 and 13*S*-LOX-1 for 678 aligned Cα (Fig. 4*B*) (44). Similar to 13*S*-LOX-1, LOX2 Ser^600^ is located on an arched helix that borders the LOX2 active site. The pLDDT values for residues located within this helix vary between 86.8 to 98.2, indicating medium to high confidence. The arched helix configuration is notably almost identical to the one observed in the AlphaFold structures of fifty-five LOX2 homologs (Fig. 4, *D* and *E*). The residue immediately adjacent to Ser^600^, Leu^601^, is highly conserved among LOXs (Table S1) as it constitutes one of the walls of the catalytic cavity and is critical to maintain the substrates in a U-shaped configuration (26). To understand the structural consequences of the phosphorylation on the Serine of the arched helix, we modeled a phosphate moiety on Ser^600^ in three distinct orientations (Fig. 4*F*). In all three cases, the phosphate moiety obstructs access to the catalytic pocket and displays steric hindrance due to the proximity of helix α2. Overall, this analysis suggests that the phosphorylation of Ser600 is likely to trigger local changes in the configuration of LOX2 as compared to the structure modeled by AlphaFold.

### Helix α2 rims the active site and appears involved in the on/off phosphorylation mechanism

Lipoxygenases with a Ser or Thr residue at the position equivalent to LOX2 Ser^600^ have a conserved basic residue located towards the N-terminal of helix α2. This residue is either a Lys located at position 10 or an Arg located at position 11 on the alignment of 13*S*-LOXs (Table S2). For 9*S*-LOXs, this residue is a Lys located at position 13 (Table S2). Those basic residues are in close proximity to the Ser^600^ phosphorylation site and could prevent substrates from accessing the catalytic pocket by locking helix α2 in proximity of the arched helix. The pLDDT values for LOX2 helix α2 are however lower on the AlphaFold model (Fig. 4*C*), ranging from 46.2 to 84.3 (low-medium confidence). Structural alignments also show that helix α2 adopts different positions in the different LOXs (Fig. 4, *D* and *E*). The low pLDDT values of residues within helix α2 (Table S2) is consistent with the crystals structures of LOXs in free or substrate-trapped conformations that also show high variability in the positioning of helix α2. This variability appears necessary to allow substrate entry into the catalytic pocket in an open conformation while catalysis occurs in a closed confirmation.

### Site-directed mutants of the Ser^600^ phosphosite suggests that its phosphorylation inhibits AtLOX2 activity

To characterize the importance of the Ser^600^ phosphosite on AtLOX2 activity, kinetic analyses were performed on the mutant (Atlox2^S600M^, Atlox2^S600D^, and Atlox2^S600A^) variants with the three substrates LA, α-LeA, and AA at pH 7.0 and 8.2 (Table 2). LOX activity was undetectable using the phosphomimics AtLox2^S600D^ with the substrates α-LeA or AA and AtLox2^S600M^ with all three substrates at either pH, 7.0 or 8.2. Low activity was obtained using LA as the substrate, but compared to AtLOX2^WT^, enzyme efficiency was greatly reduced in AtLOX2^S600D^. Due to the low specific activity, large experimental errors were obtained with *S*_0.5_ values for this phosphovariant. Taken together, these results indicate that in the phosphomimic mutants, AtLOX2 activity is severely reduced. In contrast, the phosphonull mimic Atlox2^S600A^ maintained its enzymatic activity with all three substrates. It was also interesting to note that at pH 8.2, the specific activity of the Atlox2^S600A^ was more than 1.7-fold higher than that of the WT protein with α-LeA. In addition, compared with AtLOX2^WT^, calculated values for *k*_cat_/*S*_0.5_ were more than two fold higher with Atlox2^S600A^ at pH 7.0 for α-LeA and at pH 8.2 for LA. This indicates that, in these conditions, the small hydrophobic side chain (Ala) or a small polar residue (Ser) facilitate catalytic activity in comparison to residues that are negatively charged and/or bulkier (Asp or Met). Together, these data strongly support the conclusion that phosphorylation of AtLOX2 at Ser^600^ negatively affects enzyme activity.

**Table 2:** Kinetic analyses of phosphosite variants support that Ser^600^ phosphorylation of AtLOX2 decreases enzyme activity

	AtLOX2^WT^						Atlox2^S600A^						Atlox2^S600D^	
Substrate	α-LeA		LA		AA		α-LeA		LA		AA		LA	
**pH**	7.0	8.2	7.0	8.2	7.0	8.2	7.0	8.2	7.0	8.2	7.0	8.2	7.0	8.2
***V***_***max***_ (μmol min^-1^ mg^-1^)	0.17 ± 0.03^2,b^	0.42 ± 0.08^1,a^	0.07 ± 0.01^2,c^	0.20 ± 0.01^1,b^	0.22 ± 0.02^1,a^	0.15 ± 0.03^1,b^	0.20 ± 0.04^2,a^	0.73 ± 0.25^1,a,^∗	0.13 ± 0.04^2,a^	0.35 ± 0.13^1,a,b^	0.15 ± 0.03^1,a^	0.15 ± 0.05^1,b^	0.015 ± 0.005	0.007 ± 0.003∗
***S***_**0.5**_ (μM)	61.3 ± 5.8^1,a^	108.3 ± 17.2^1,a^	26.7 ± 2.5^2,c^	82.6 ± 6.3^1,b^	38.5 ± 1.8^1,b^	40.7 ± 7.2^1,c^	34.60 ± 13.65^2,a^	141.43 ± 56.46^1,a^	32.39 ± 14.59^2,a^	68.57 ± 14.44^1,a^	29.87 ± 3.74^1,a^	54.70 ± 16.47^1,a^	52.8 ± 54.3	37.0 ± 25.6
***k***_**cat**_ (s^-1^)	0.28 ± 0.04^2,b^	0.70 ± 0.13^1,a^	0.12 ± 0.02^2,c^	0.33 ± 0.02^1,b^	0.37 ± 0.03^1,a^	0.24 ± 0.05^1,b^	0.33 ± 0.06^2,a^	1.22 ± 0.42^1,a,^∗	0.22 ± 0.07^2,a^	0.58 ± 0.21^1,a,b^	0.24 ± 0.06^1,a^	0.26 ± 0.09^1,b^	0.02 ± 0.01	0.01 ± 0.05∗
***k***_**cat**_**/*S***_**0.5**_ (s^-1^ mM^-1^)	4.62 ± 0.37^1,b^	5.58 ± 0.19^1,a^	4.56 ± 0.70^1,b^	4.07 ± 0.27^1,b^	9.58 ± 0.71^1,a^	5.95 ± 1.00^2,a^	10.46 ± 3.58^1,a,^∗	8.76 ± 1.17^1,a^	7.14 ± 0.001^1,a^	8.33 ± 0.01^1,a,^∗	8.13 ± 1.05^1,a^	4.69 ± 0.74^2,b^	0.72 ± 0.41∗	0.35 ± 0.09∗
***h***	2.7	1.9	3.4	1.6	2.1	2.1	1.9	2.0	1.8	1.8	2.8	2.2	1.6	2.7

AtLOX2 saturation kinetics were compared between WT (AtLOX2^WT^) and variants that mimic phosphorylation (Atlox2^S600D^ and Atlox2^S600M^) or a phosphonull mimic that cannot be phosphorylated (Atlox2^S600A^) using the substrates α-linolenic acid (α-LeA), linoleic acid (LA), and arachidonic acid (AA) at pHs 7.0 and 8.2, respectively, representing the chloroplast stromal pH in the dark and light. Values are the mean ± SD for least three temporal replicates.

Statistical differences between WT and variant lines for pH and substrate were determined by ANOVA. For each enzyme (AtLOX2^WT^ or Atlox2^S600A^ or Atlox2^S600D^), differences due to substrates and pH combinations are shown by numbers. For each specific enzyme and substrate combination, differences due to pH are shown by an asterisk. For each specific substrate and pH combination, differences between enzymes are shown by alphabetical letters. AtLox2^S600M^ activity for all three substrates and Atlox2^S600D^ activity of α-LeA and AA were below detection limits of the assays (*e.g.*, 0.03 nmol/min for α-LeA). Statistical differences for Hill’s constant were not determined.

## Discussion

Jasmonates are important phytohormones in plant defense, particularly against chewing insect pests and necrotrophic pathogens (2). They are also involved in plant development (1, 2, 3, 4). Jasmonate biosynthesis, signaling, and perception is posttranslationally regulated (19). In arabidopsis, AtLOX2 is a key enzyme in jasmonate biosynthesis. In this species, phosphorylation of AtLOX2 Ser^600^ was observed in undamaged plants (31). In contrast, a Ser^600^-dephosphorylated form of the enzyme was present in damaged plants. Based on these data, we formulated the hypothesis that phosphorylation on Ser^600^ could play a role in regulating AtLOX2 enzyme activity. Thus, dephosphorylation of the constitutive enzyme would be leading to higher jasmonate biosynthesis in damaged plants.

To test this hypothesis, we produced a recombinant mature AtLOX2 protein by removing the N-terminal targeting peptide from the coding sequence. The purified recombinant protein had the expected molecular mass of 98.7 kDa. A minor, closely migrating band was found below the band corresponding to AtLOX2^WT^ and its site-directed mutants. Such double banding pattern was previously seen with recombinant pea seed LOX (45). In the present study, the fact that the two bands were recognized by a commercial anti-LOXC immune serum that cross reacts with AtLOX2 (38) suggests the occurrence of limited proteolysis of AtLOX2. Alternatively, but less likely, this type of pattern can also be caused by DTT-resistant intermolecular or intramolecular disulfide bridge(s) causing a shift in protein migration (46). Interestingly, previous observations suggested a possible redox regulation of AtLOX2, which contains several conserved Cys residues (47).

The recombinant AtLOX2^WT^ was further analyzed by LC-MS/MS. No peptide carrying a phosphorylated Ser^600^ residue was found, confirming that the protein was not phosphorylated by a promiscuous *E. coli* protein kinase during its production.

In this research, we studied the kinetic properties of recombinant AtLOX2^WT^ as well as the phosphonull mutant Atlox2^S600A^ and two phosphomimics, Atlox2^S600D^ and Atlox2^S600M^. Our data demonstrate that enzyme activity is lower in the phosphomimic mutants, suggesting that AtLOX2 catalysis is negatively affected by phosphorylation at this site.

In leaves, AtLOX2 is localized in the chloroplast stroma (48, 49, 50). During the day, in an actively photosynthesizing leaf cell, the stromal pH increases from neutral to an alkaline pH value around 8, as the photosynthetic electron transport chain results in the movement of protons from the stroma to the thylakoid lumen (51, 52). Foliar jasmonate levels also fluctuate in a day:night cycle, with the highest levels being present during the afternoon (53, 54, 55). To study the effect of pH on AtLOX2^WT^ enzyme kinetics, the assays were carried out over six pH values ranging from pH 7.0 to pH 8.8, encompassing the physiological pH range of chloroplast shifting from dark to light.

In higher plants, α-LeA and LA are the predominant PUFAs found in leaves, respectively, representing 50% and 14% of total fatty acids (34). α-LeA is the precursor to defensive oxylipins such as OPDA and JA-Ile (56). In the present work, AtLOX reaction rates were recorded under steady state conditions. Recombinant AtLOX2^WT^ exhibit positive cooperative binding with respect to its endogenous substrates, α-LeA and LA, as well as with AA (Fig. 1). Even though previous studies of native or recombinant plant LOXs have reported Michaelis–Menten behavior for these enzymes (50, 57, 58, 59, 60), cooperative binding has also been previously observed with α-LeA (45, 61) or LA (62) as substrates. AtLOX2^WT^ has the highest activity with α-LeA as the substrate over the tested pH range, in comparison to LA (Table 1). This latter observation agrees with previous studies in which the specificity of partially purified arabidopsis AtLOX2 for various substrates was compared (27, 50). Despite the fact that substrate saturation experiments show the highest *k*_cat_ values when α-LeA is the substrate, affinity for α-LeA was lower than for LA or AA. For the physiological substrates α-LeA and LA, these results reflect a higher conversion rate with α-LeA but better binding affinity with LA. Similar observations were seen for olive- and poppy-derived LOX enzymes (58, 63).

Optimum pH for LOXs in higher plants varies depending on isoforms studied and substrates used, with highest activity values reported in a wide pH range of 6 to 9 (35, 58, 59, 61, 64). We found that, even though the AtLOX2^WT^ reaction rate increases with pH for both α-LeA and LA as substrates (Table 1), its substrate affinity decreased concomitantly. Therefore, for both α-LeA and LA substrates, there is little change in catalytic efficiency over this pH range. A recent study characterized recombinant arabidopsis AtLOX2 activity (50). While we share some of their observations concerning substrate preference, certain aspects of our results differ from theirs. In particular, these authors observed a more acidic pH optimum value and Michaelis–Menten kinetics rather than cooperative behavior for AtLOX2. These differences could possibly be attributed to some or all of the following factors: (i) the use of different bacterial expression system; (ii) differences in the removal of the N-terminal targeting peptide from the recombinant protein in the two studies and (iii) the higher degree of purity of our AtLOX2 preparation. The first two factors could impact protein folding and kinetic properties, whereas differences in purity would affect the presence of contaminating competing enzymes or protein effectors in activity assays.

With a few rare exceptions, AA is not found in seed plants and is, therefore, not considered a natural substrate for plant LOXs (34). The reaction rate of AtLOX2^WT^ for AA remains low over the pH range but this enzyme has a higher substrate affinity for AA than α-LeA (Table 1). Thus, over the pH range, the catalytic efficiency of AtLOX2^WT^ for AA is similar to α-LeA. Given that AA is not an endogenous substrate in higher plants, this is not considered to be a competing reaction.

A previous report identified the phosphorylation of AtLOX2 on Ser^600^ (31). AtLOX2 was shown to be phosphorylated constitutively and dephosphorylated in response to mechanical damage. This differential phosphorylation of AtLOX2 could participate in a regulatory mechanism over the jasmonate biosynthesis pathway. In the present work, phosphovariants of Ser^600^ were generated and characterized to elucidate the role of this phosphosite on LOX activity. A change of Ser to Ala was done to mimic the dephosphorylated state of AtLOX2, whereas changing Ser to Asp mimics the bulkiness and presence of a negative charge of phosphorylated Ser. The Ser to Met mutation was generated to approximate the space occupied by the side chain of an Asp residue or a phosphor-Ser without the presence of a negative charge.

As Ser^600^ lies at a critical position within the AtLOX2 catalytic pocket, we envision several ways in which changes at this position could affect AtLOX2 activity (Fig. 4). First, Ser^600^ phosphorylation could perturb the structure or dynamics of the arched helix by promoting interactions with neighboring residues (*e.g.* Arg^596^). Second, phosphorylation of Ser^600^ could disturb the structure or dynamics of the adjacent α2 through steric effects. Indeed, in the structure of soybean 13*S*-LOX-1, access to the catalytic pocket is restricted by helix α2. Residues on this helix (residues 261–267 corresponding to residues 318–324 in AtLOX2) undergo nanosecond backbone fluctuations at alkaline pH, thus identifying helix α2 as a probable point of entry for the substrates (65). Given that Ser^600^ lies at the interface between the arched helix and helix α2, Ser^600^ phosphorylation therefore appears as a possible mechanism to regulate substrate entry into the catalytic pocket, either by promoting interaction with a basic residue within helix α2 and/or by directly preventing the substrate from accessing the catalytic pocket. The decreased LOX activity observed when Ser^600^ is substituted by an Asp or a Met residue is consistent with this mechanism. Third, structural comparison of the AlphaFold model of AtLOX2 with a complex between 8R-LOX and a 20C PUFA suggests a close proximity of Ser^600^ with the carboxylic acid head group of the PUFA. Although the exact positioning of substrates within the AtLOX2 catalytic pocket is still unknown, this comparison suggests that Ser^600^ phosphorylation could antagonize substrate binding through electrostatic repulsion with the carboxylic group of the substrate. Overall, our analyses thus suggest that Ser^600^ is located at a critical position within AtLOX2 structure and that phosphorylation of this residue is likely to affect the catalytic activity of AtLOX2.

The phospho-null variant, Atlox2^S600A^, exhibits similar kinetics to the WT enzyme, which is dephosphorylated (Table 2). In contrast, AtLOX activity is not detectable in the phosphomimics, Atlox2^S600D^ and Atlox2^S600M^. The only exception was an extremely low specific activity found with Atlox2^S600D^ in the presence of LA. In this latter case, the *V*_*max*_ and the catalytic efficiency of the recombinant Atlox2^S600D^ enzyme is lower than AtLOX^WT^ or Atlox2^S600A^ and close to background levels. It is of interest that the change in amino acid did not affect substrate affinity. These data support the role of phosphorylation in the regulation of AtLOX2, where the constitutive, phosphorylated enzyme has minimal activity. Upon wounding or chewing herbivore attack, dephosphorylation results in higher activity leading to jasmonate biosynthesis.

Our analyses also showed that, in plant LOXs identified to be involved in defense, the arabidopsis phosphoSer site may be a Ser, Thr, or Ala (Fig. S3 and Table S1). This raises the intriguing possibility that LOXs containing Ser or Thr (corresponding to position 600 in arabidopsis) have evolved to be modified by phosphorylation and, therefore, sensitive to stress conditions with an on/off effect on activity. Other defense-associated LOXs contain the nonphosphorylable Ala at position 600 implying that this protein is constitutively active but perhaps regulated at the transcriptional level. In arabidopsis leaves, AtLOX2 and AtLOX6 are 13*S*-LOXs that play the largest role in wound-induced jasmonate biosynthesis and signaling with contributions from AtLOX3 and AtLOX4 (28, 29). AtLOX3 and AtLOX6 have a highly conserved Ser that corresponds to AtLOX2 Ser600 and AtLOX4 has a Thr, suggesting that the activity of these enzymes may be regulated by phosphorylation. In contrast, arabidopsis 9*S*-LOXs AtLOX1 and AtLOX5 have Ile or Val in this position. It is tempting to speculate that LOX with a Ser or Thr present are expressed constitutively and regulated posttranslationally by phosphorylation/dephosphorylation. This allows rapid activation and dynamic regulation of the pathway in response to a stress. Indeed, the “instant” increase in jasmonates in response to wounding precludes gene expression (6). In contrast, proteins that contain Ala, Ile, or Val at this site suggests multiple levels of AtLOX regulation. Such a hypothesis will need to be experimentally verified. Nonetheless, our data strongly support the conclusion that phosphorylation of AtLOX2 at Ser^600^ negatively affects enzyme activity.

**Figure 5:**
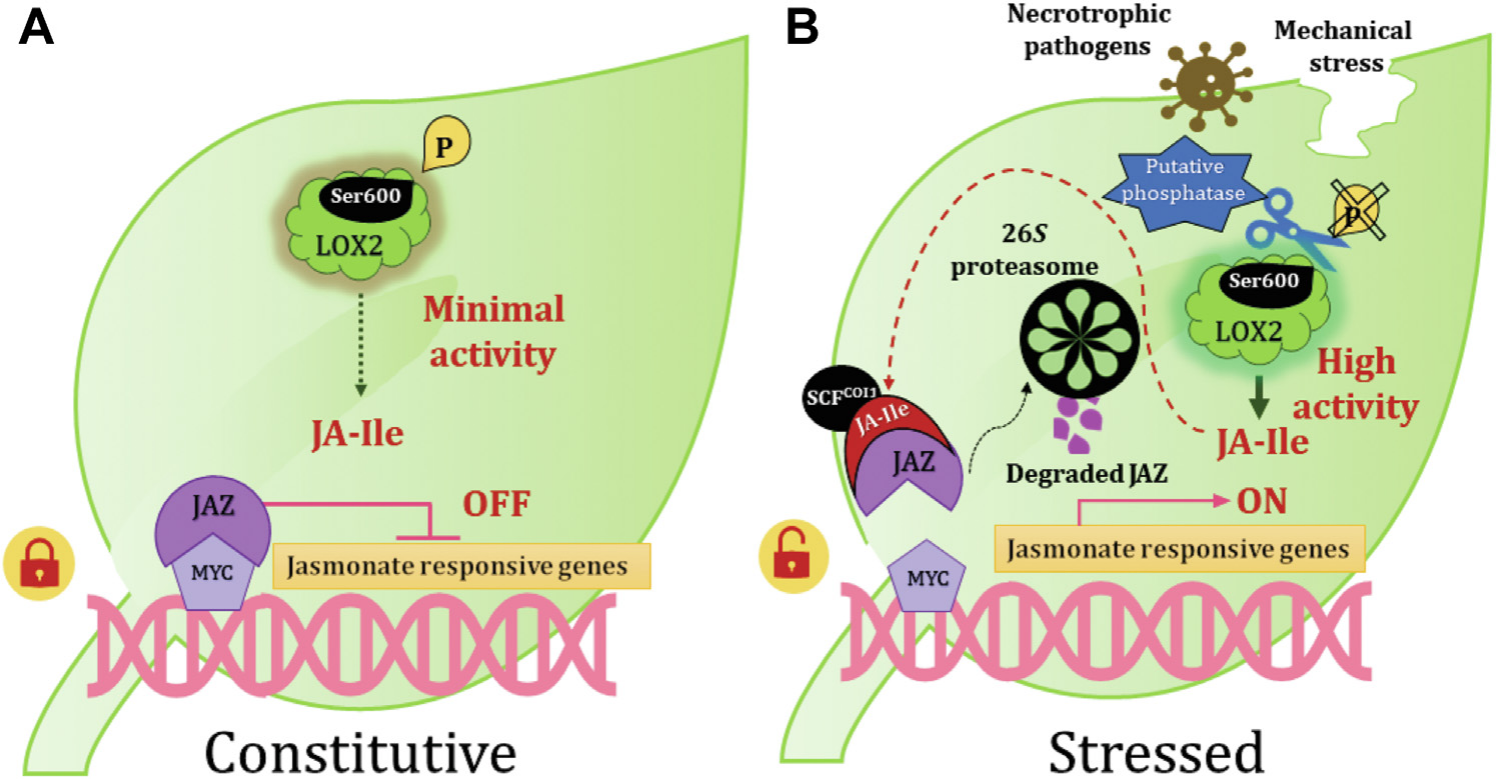
**A proposed model integrating the effects of LOX2 Ser**^**600**^**phosphorylation status on the jasmonate signaling pathway.***A*, under constitutive, unstressed conditions, LOX2 Ser^600^ is present in a phosphorylated state, in which the enzyme has minimal activity, resulting in low JA-Ile levels. In this state, the MYC transcription factor is repressed by Jasmonate-Zim domain (JAZ) proteins along with other corepressors, preventing the transcription of jasmonate-responsive genes. *B*, under stressed conditions (*e.g.* following necrotrophic pathogen attack or mechanical damage), LOX2 Ser^600^ is dephosphorylated by a putative phosphatase, leading to high LOX2 activity. The ensuing JA-Ile outburst results in the ubiquitination of JAZ repressor proteins by the SCF^COI1^ ubiquitin complex leading to a 26*S*-proteasome–mediated degradation. Consequently, the MYC transcription factor is derepressed, allowing jasmonate-responsive gene expression. JA, jasmonic acid; JA-Ile, (+)-7- iso-jasmonoyl-isoleucine; LOX, lipoxygenase.

Our results enable us to propose a working model integrating the differential phosphorylation of AtLOX2 Ser^600^ as a key mechanism for the regulation of AtLOX2 activity and, thus, jasmonate biosynthesis (Fig. 5). In this model, under constitutive (unstressed) conditions, LOX2 is phosphorylated on Ser^600^, and the enzyme has minimal activity. In this state, synthesis of JA-Ile is predicted to be low and the MYC transcription factor is repressed by JAZ proteins and other corepressors, preventing the transcription of jasmonate-responsive genes. Under stress generated by necrotrophic pathogens or mechanical damage, LOX2 Ser^600^ is dephosphorylated by a putative phosphatase. As a result, LOX2 activity increases JA-Ile biosynthesis. Consequently, the ubiquitination and the degradation of the JAZ repressors *via* the 26*S*-proteasome occurs. In the absence of repressors, jasmonate-responsive genes under the control of the MYC transcription factor are expressed. This model involves the participation of putative protein kinase(s) and phosphatase(s) acting on LOX2 Ser^600^. These enzymes are key elements for understanding the stress-regulation of jasmonate synthesis but have not been currently characterized. However, the availability of data concerning chloroplastic protein kinases, protein phosphatases, and their targets is steadily increasing (66, 67, 68, 69, 70, 71). Such data should facilitate the search for the relevant protein modifiers.

It is interesting that this posttranslational modification is taken advantage of by beet armyworm (*Spodoptera exigua*) caterpillars feeding on the plant. Beet armyworm caterpillars manage to circumvent plant defenses though effectors found in their labial salivary secretions (72, 73, 74). In response to mechanical wounding or herbivory by caterpillars with impaired labial salivary secretions, AtLOX2 is dephosphorylated leading to robust jasmonate responses (31). However, in arabidopsis attacked by caterpillars with intact labial salivary secretions, AtLOX2 remains phosphorylated and reduced defense responses are observed (31, 72, 73, 74). Given the importance of Ser^600^ for the regulation of AtLOX2 revealed by the present work, the effect of labial salivary secretions on AtLOX2 phosphorylation status identifies a key functional element in *S. exigua* strategy to undermine plant resistance.

## Conclusion

Jasmonates are key defense signaling molecules in plants (1, 2, 4). As such, understanding the regulation of jasmonate biosynthesis is of great importance since it may lead to the enhancement of plant resistance. Our previous data showed that AtLOX2 is constitutively phosphorylated in undamaged plants that have basal jasmonate levels (31, 72, 73, 74). The present study characterizing the kinetic properties of AtLOX2 phosphomimic and phosphonull mutants leads us to conclude that phosphorylated AtLOX2 has minimal activity. Dephosphorylated AtLOX2 was found in damaged plant leaves that have higher jasmonate levels (31, 72, 73, 74). Thus, we further hypothesize that in response to stress, such as mechanical wounding or recognition of necrotrophic pathogens, a phosphatase removes the phosphate group on Ser^600^, activating the enzyme and leading to jasmonate biosynthesis. For the endogenous plant substrates, α-LeA and LA, the reaction rate is higher at pH 8.2 than 7.0 respectively corresponding to prevailing conditions in light and dark. However, overall catalytic efficiency was not affected by pH (Table 1). Thus, in addition to the phosphorylation status, substrate availability may also be an important component of the control over AtLOX2 activity in light *versus* dark conditions.

## Experimental procedures

### Biological materials and chemicals

Chemicals and reagents used were of analytical grade and were obtained from Sigma chemical or Thermo Fisher Scientific. α-LeA, LA, and AA were purchased from Sigma-Aldrich. Ni-NTA agarose was purchased from Thermo Fisher Scientific.

### Sequence analysis and modeling of AtLOX2

Fifty-five LOX amino acid sequences from organisms ranging from plants (monocots, dicots) and animals were obtained from NCBI and aligned using Geneious Prime 2022.0.1 (listed in Table S1). These sequences were used to generate a phylogenetic tree. In addition, we also performed alignments on the protein sequence that was trimmed to a 21 amino acids sequence (10 amino acids before and 10 amino acids after) surrounding the putative phosphorylated Ser present at position 600 in AtLOX2 to identify conserved amino acids surrounding the Ser. A phylogenetic analysis of these 21 amino acids was conducted using MEGA 11.0 with neighbor-joining and bootstrap values from 1000 replicates.

The structure of arabidopsis LOX2 was obtained through the AlphaFold Protein Structure Database (accession number P38418) that used AlphaFold Monomer v3.0 for structure prediction (42, 43, 75). To verify that this structure was not biased towards a specific PDB template, we also modeled LOX2 using ColabFold with the option not to use PDB templates (76). The resulting structure was very similar to the one present in the AlphaFold Protein Structure Database with a RMSD of 0.76 Ǻ over 695 aligned Cα (corresponding to residues 202–896). Given the strong similarity between these models, we used the structure from the AlphaFold Protein Structure Database for all subsequent analyses. The identification of structural neighbors of LOX2 was performed using Dali (44). A PUFA substrate was positioned within the active site of LOX2 by aligning the structure of *Plexaura homomalla* 8R-LOX (pdb 4QWT), that contains a PUFA, with the structure of the catalytic domain of LOX2 generated using AlphaFold. The positioning of iron within the active site of LOX2 was similarly obtained by aligning the structure of soybean 13*S*-LOX-1 (pdb 1YGE), that contains iron, with the structure of the catalytic domain of LOX2 generated using AlphaFold. In Figure 4, *C*, *D* and *E*, the level of confidence for the residues of the arched helix and helix α2 is represented by their pLDDT values as determined by AlphaFold: very high above 90 (blue), confident between 70 and 90 (pale blue), low between 50 and 70 (yellow), and very low below 50 (orange). The structures of 13S and 9S plants’ LOXs depicted in Figure 4, *D* and *E* were obtained through the AlphaFold Protein Structure Database (accession numbers reported in the Tables S1 and S2). The visualization software PyMOL (https://pymol.org/2/, The PyMOL Molecular Graphics System, Version 2.5.2 Schrödinger, LLC.) was used to analyze the structure, perform structural alignments, add a phosphate moiety to Ser^600^ of LOX2, and to generate figures.

### Site-directed mutagenesis of AtLOX2 and generation of constructs for heterologous expression

The WT *AtLOX2*-coding sequence obtained from the RIKEN institute (RAFL09-06-O22) was inserted into pBluescript and modified by site-directed mutagenesis (Norclone Biotech Laboratories) to generate *Atlox2*^*S600D*^*, Atlox2*^*S600M*^, or *Atlox2*^*S600A*^ in which the codon for Ser^600^ is respectively replaced by codons for Asp, Met, or Ala. The substitution of Ala introduces a nonphosphorylatable version of the protein, whereas the Asp mimics the phosphoSer. In addition, the Met substitution introduces a bulky residue but that, in contrast to Asp, does not have a negative charge. From these vectors, the AtLOX2^WT^ and the mutated coding sequences were amplified by PCR using primers that introduced *attB* sites at the 5′-end of the forward primer and cloned into a pCR8/GW/TOPO Gateway entry vector. To avoid issues with the heterologous expression of proteins carrying an N-terminal targeting peptide (77, 78), the primers 5′-GCTAATATTGAACAAGAAGGTAACACAG-3′ and 5′ CGAACCGAACAGGCTTATGT 3′ were used to generate constructs of AtLOX2^WT^ and Atlox2 variants without the predicted chloroplast transit peptide (N-terminal amino acids 1–56). After digestion with *EcoR*I, the inserts were ligated into the pProEX HTb expression vector, predigested with *Ehe*I and *EcoR*I. The final constructs were transformed into *E. coli* HB101l^-^ and, after confirmation of DNA sequence, were used to produce soluble recombinant AtLOX2^WT^ and the nonphosphorylable (Atlox2^S600A^), the phosphomimic (Atlox2^S600D^), and the Atlox2^S600M^ variant.

### Heterologous protein production, purification, electrophoresis, and immunoblot analysis

For recombinant protein production, *E. coli* HB101l^-^ cells harboring the constructs encoding recombinant His_6_-AtLOX2^WT^ or its variants (Atlox2^S600A^, Atlox2^S600D^, Atlox2^S600M^) were grown in 300 ml of LB media containing ampicillin (100 μg/ml) at 37 °C. Upon reaching an absorbance of 0.5 to 0.6 at 600 nm, IPTG (0.6 mM final concentration) was added to the cultures which were then grown overnight at 17 °C, with shaking at 250 rpm. Bacterial cells were harvested by centrifugation at 5000*g* for 15 min at 4 °C. The pellets were frozen and kept at −80 °C until used.

For protein purification, frozen pellets were resuspended in 25 ml of extraction buffer (300 mM NaCl, 50 mM NaH_2_PO_4_, 10 mM imidazole, 0.1% Triton X-100 (v/v), 1 mM PMSF, pH 8.0) and extracted using a French press at 18,000 psi. The extract was then centrifuged at 20,000*g* for 30 min at 4 °C. Recombinant proteins were purified by immobilized metal affinity chromatography using Ni-NTA agarose in an open column essentially as described previously (79). Briefly, the protein extract was allowed to bind batch wise (60 min at 4 °C with gentle shaking) to the Ni-NTA agarose matrix (1 ml settled bed volume, previously equilibrated in 300 mM NaCl, 10 mM imidazole, and 50 mM NaH_2_PO_4_, pH 8.0). After this step, the slurry was poured into a disposable column (6.5 cm height x 1 cm diameter) followed by a wash with 4 ml of wash buffer (300 mM NaCl, 20 mM imidazole, 50 mM NaH_2_PO_4_, pH 8.0). The bound protein was eluted by increasing imidazole concentration of the extraction buffer to 250 mM. Elution fractions of 1 ml were collected and a 5 μl aliquot was denatured in SDS-PAGE sample buffer (80) in the presence of 25 mM DTT. The presence of recombinant protein in the elution fractions was observed by analyzing the denatured aliquot of each fraction on a 10% (w/v) SDS-PAGE, followed by Coomassie blue staining (0.1% (w/v) Coomassie blue R-250, 50% (v/v) methanol, 10% (v/v) acetic acid). The three or four fractions containing the highest amount of recombinant protein were pooled and subjected to dialysis at 4 °C in a 30 mM sodium phosphate buffer, pH 7.5 containing 50 mM NaCl. The dialyzed protein was centrifuged at 20,000*g* for 30 min at 4 °C to remove insoluble material and stored at −20 °C in 50% (v/v) glycerol. Protein concentration was determined using the Bradford assay kit (Bio-Rad, Mississauga) and bovine serum albumin as the standard (81). Activity of the stored protein was stable for at least 1 month at −20 °C without loss of activity. The purified protein was analyzed by Western blot using an anti-LOX-C rabbit polyclonal antibody (Catalog #AS07258) known to cross react with AtLOX2 (38). Detection was done using a goat anti-rabbit IgG conjugated to alkaline phosphatase (82).

### Analysis of recombinant AtLOX2^WT^ by MS

Fifty micrograms of purified recombinant AtLOX2^WT^ were vortexed in 50 mM ammonium bicarbonate containing 10 mM Tris(2-carboxyethyl)phosphine hydrochloride for 1 h at 37 °C. The sample was then alkylated by treatment with 55 mM chloroacetamide, followed by vortexing for 1 h at 37 °C. Protein digestion was performed by adding 1 μg trypsin and incubating for 8 h at 37 °C. After drying, tryptic peptides were solubilized in 5% (v/v) acetonitrile and 0.2% (v/v) formic acid and separated using a custom-made reversed-phase column (150 μm x 200 mm) on an EASY-nLC 1000 liquid chromatograph system connected to an Orbitrap Fusion (Thermo Fisher Scientific). Chromatographic separation used a linear gradient from 10 to 30% (v/v) acetonitrile in 0.2% (v/v) formic acid over 56 min at a flow rate of 600 nl/min. Full MS spectra were acquired at a resolution of 120,000 followed by MS/MS spectra acquisition on the most abundant multiply charged precursor ions for a maximum of 3 s. MS/MS data were acquired using collision-induced dissociation at a collision energy of 30%. The PEAKS X pro software (https://www.bioinfor.com/peaks-xpro/, Bioinformatics Solutions) and a AtLOX2 database (1 entry) were used to process the data, respectively, using precursor and fragment ions mass tolerances of 10 ppm and 0.3 Da and taking into account a fixed carbamidomethyl modification on Cys residues. Other analysis settings included variable posttranslational modifications: Met oxidation, Gln and Asn deamidation, Ser, Thr and Tyr phosphorylation, N-terminal acetylation. The Scaffold 5.1.0 software (https://www.proteomesoftware.com/products/scaffold-5, Proteome Software, Inc) was used for data visualization (protein threshold, 99%, with at least two peptides identified and a false-discovery rate of 1% for peptides).

### Enzyme activity and kinetic analyses

The kinetic analyses of AtLOX2^WT^ and the Atlox2 variants were carried out using spectrophotometric enzyme assays at 30 °C with a SpectraMax i3X microplate spectrophotometer controlled by the SoftMax Pro 7.0 Software (https://www.moleculardevices.com/, Molecular Devices, San Jose, CA). The reaction mixture (250 μl total volume) contained 10 μg of affinity-purified recombinant AtLOX2^WT^ or variant enzymes (Atlox2^S600A^, Atlox2^S600D^, Atlox2^S600M^) in 100 mM Tris–HCl buffer adjusted to the specified pH. The buffer was fully aerated by constant stirring to ensure saturation with ambient air (83). Based on the solubility of dissolved oxygen, the concentration of oxygen in assays was approximately 235 μM. The assays for recombinant AtLOX2^WT^ were carried out over a pH range from 7.0 to 8.8, whereas the variant Atlox2 kinetics were only determined at pH 7.0 and 8.2, which reflects the two extreme pH conditions in the chloroplast stroma during dark or light conditions, respectively (51, 52). Substrates tested for saturation kinetics were α-LeA, LA, and AA. Substrate stocks were dissolved in 95% (v/v) dimethyl sulfoxide and used to generate a range of substrate concentrations (0–350 μM final concentration). To initiate the reaction, the purified enzyme was added to the reaction mixture containing the substrate in the wells of UV-compatible microplate. The change in absorbance was measured at 234 nm and recorded at 15 s intervals over 10 min. Enzyme activity was calculated from steady state velocities. Background was corrected by subtracting the activity in no-substrate wells containing dimethyl sulfoxide. The following molar extinction coefficients were used to calculate activities: for α-LeA and AA: 23,000 M^−1^ cm^−1^ (84, 85) respectively; for LA: 28,000 M^−1^ cm^−1^ (86). The detection limit of these assays was approximately 0.03 nmol/min. Kinetic parameters were calculated by the SigmaPlot 12.5 software (https://systatsoftware.com/sigmaplot/, Systat Software Inc, Palo Alto, CA). Values of apparent *V*_max_ (enzyme velocity at saturating substrate concentration), *S*_0.5_ (half saturating substrate concentration, a measure of enzyme substrate affinity for cooperative enzymes), and Hill constant (*h*) (measure of cooperativity in substrate binding) were calculated by using nonlinear regression for sigmoidal curves. *k*_cat_ (measure of the number of catalytic cycles carried out per unit of time) was calculated assuming one active site per enzyme subunit. Specific activity is expressed in unit (U) per mg of protein. One U of AtLOX2 activity corresponds to the production of 1 μmol of (13*S*)-hydroperoxy-(9*Z*,11*E*)-octadecadienoate per minute.

### Statistical analysis

All protein preparations were temporally repeated and independently analyzed at least 3 times. For the WT enzyme kinetics, significant differences were determined by 2-factor ANOVA (factors: substrate, pH) using SPSS (V. 27) followed by a Tukey HSD *post hoc* test to identify differences (*p* ≤ 0.05). For the characterization of the effect of site-directed mutagenesis on enzyme activity, significant differences in comparison to WT were determined by a 3-factor ANOVA (factors: genotype, substrate, pH) followed by a Tukey HSD *post hoc* test to identify differences (*p* ≤ 0.05). Where there was an interaction effect, a 1-factor ANOVA followed by Tukey HSD was used to identify differences.

## Data availability

All data are contained within the article. All the plasmids and bacterial strains described in this paper are available upon request to the corresponding authors.

## Supporting information

This article contains supporting information (10, 27, 28, 36, 41, 58, 60, 64, 72).

## Conflict of interest

The authors declare that they have no conflicts of interest with the contents of this article.
