# Supplementary material for: Pseudophosphorylation of Arabidopsis jasmonate biosynthesis enzyme lipoxygenase 2 via mutation of Ser600 inhibits enzyme activity
Source: J Biol Chem. 2023 Jan 10;299(3):102898. doi: 10.1016/j.jbc.2023.102898 (PMC9947334; doi:10.1016/j.jbc.2023.102898)
Supplement: Supplemental figures [file mmc1.docx]

Supplemental material to:

**Pseudophosphorylation of *Arabidopsis* jasmonate biosynthesis enzyme lipoxygenase 2 via mutation of Ser^600^ inhibits enzyme activity**

Diljot Kaur^1,2^, Sonia Dorion^2^, Souleimen Jmii^3^, Laurent Cappadocia^3^, Jacqueline C. Bede^1^ and Jean Rivoal^2^

1. Department of Plant Science, McGill University, 21,111 Lakeshore, Ste-Anne-de-Bellevue, QC, H9X 3V9, Canada
2. Institut de Recherche en Biologie Végétale, Université de Montréal, 4101 Sherbrooke est, Montréal, QC, H1X 2B2, Canada
3. Département de Chimie, Université du Québec à Montréal, Montréal, QC, H2X 3V7, Canada

**
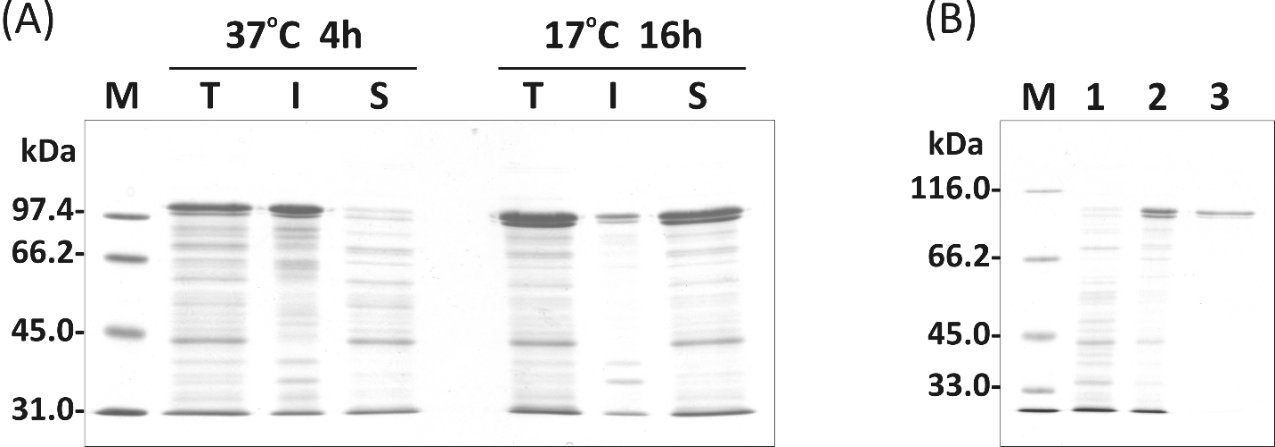
**

**Supplemental Figure 1.** **SDS-PAGE analysis of conditions for soluble recombinant** **LOX2^WT^ protein expression and purification.** A. Optimization of induction conditions for soluble protein extraction. Total (T), insoluble pellet (I) and soluble supernatant (S) protein fractions after induction by isopropyl β-D-1-thiogalactopyranoside (IPTG) at either 37°C for 4 hr or 17°C overnight (O/N). B. Purification of LOX2^WT^ . SDS-PAGE of protein aliquots from uninduced culture (lane 1), IPTG-induced culture at 17^o^C (lane 2) and eluted fraction after Ni-NTA column purification (lane 3). The purified protein migrates at ~98.7 kDa. The lanes marked M in panels A and B show the protein molecular weight markers identified on the left side of the gels.

**
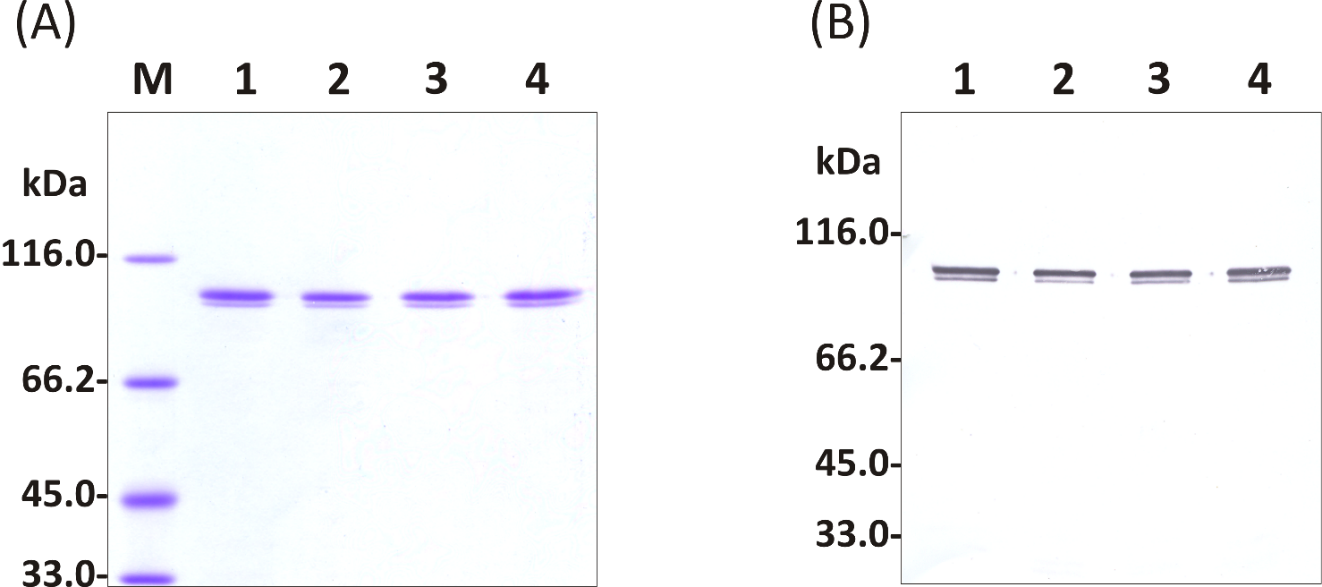
**

**Supplemental Figure 2. SDS PAGE and immunoblot analysis of purified recombinant LOX2^WT^ and variants.** LOX2^WT^ (lane 1), lox2^S600D^ (lane 2), lox2^S600M^ (lane 3) and lox2^S600A^ (lane 4) were purified by Ni-NTA chromatography. A. One μg of pooled purified protein fraction was separated on a 10% (w/v) SDS-polyacrylamide gel and visualized by Coomassie staining. B. Western blot analysis of the same proteins (25 ng) using Agrisera’s anti-LOX-C antibody. The running position of molecular weight markers (M) is indicated on the left side of the panels.


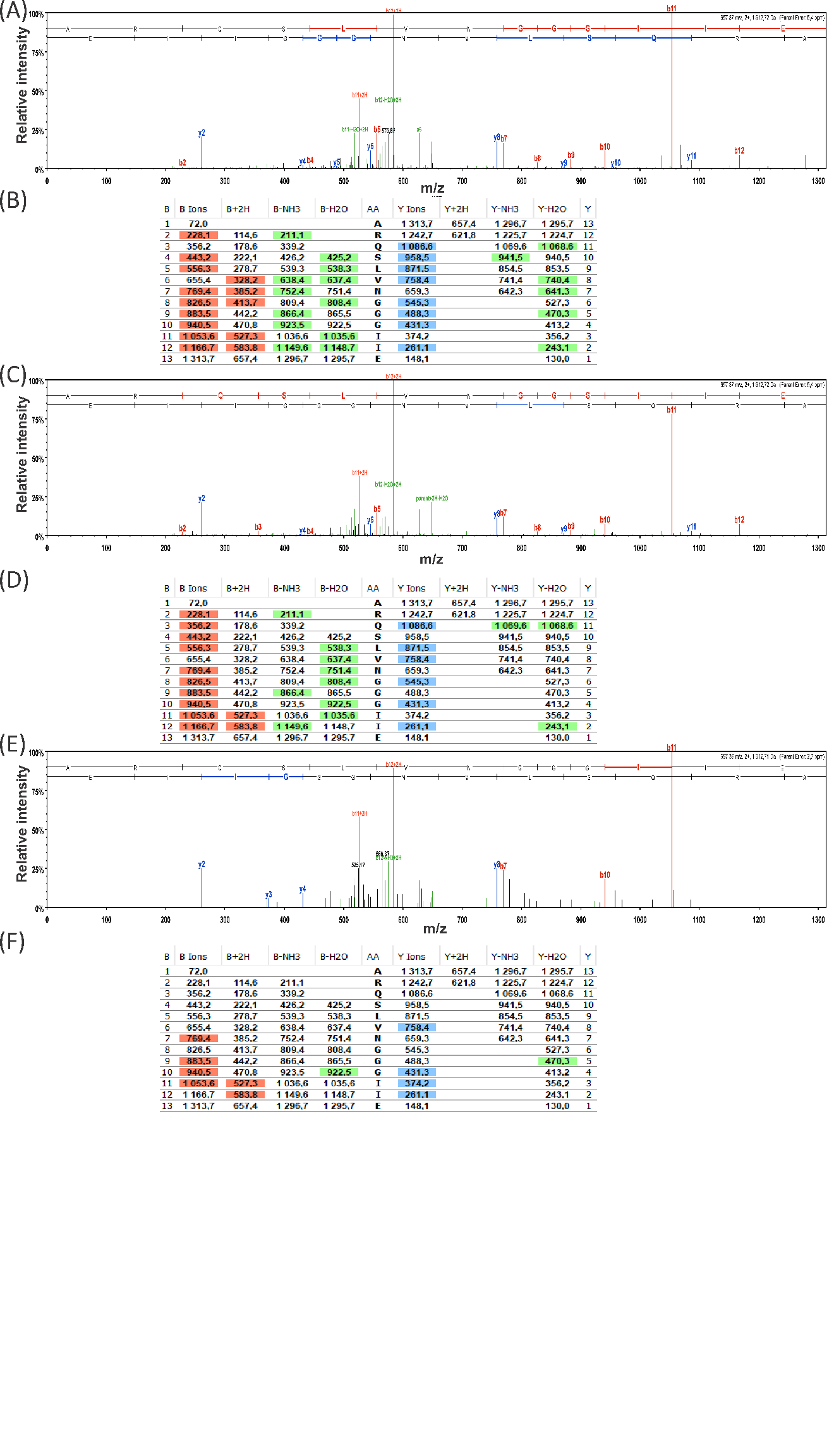


**Supplemental Figure 3 (continued)**

**
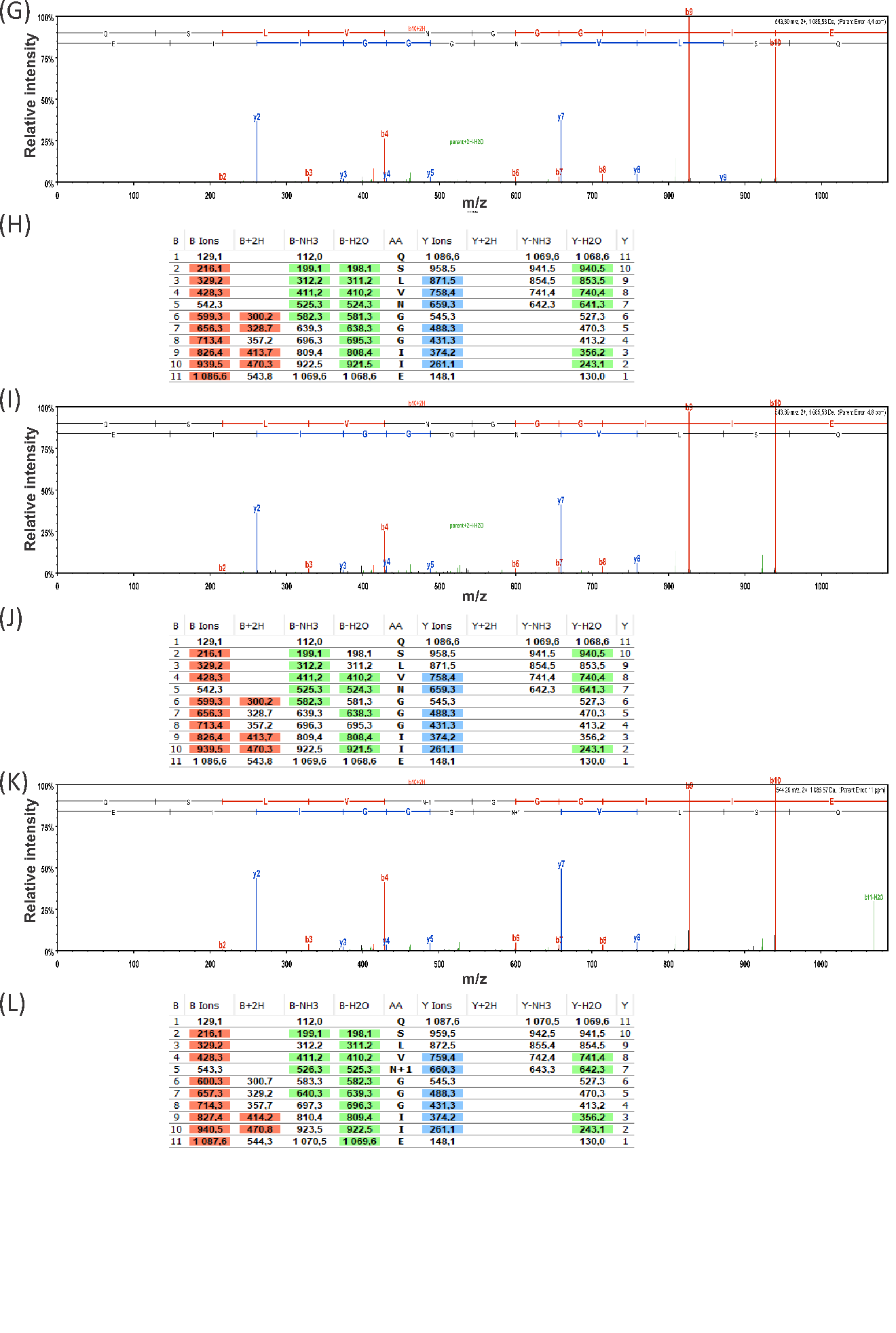
**

**Supplemental Figure 3 (continued)**

**
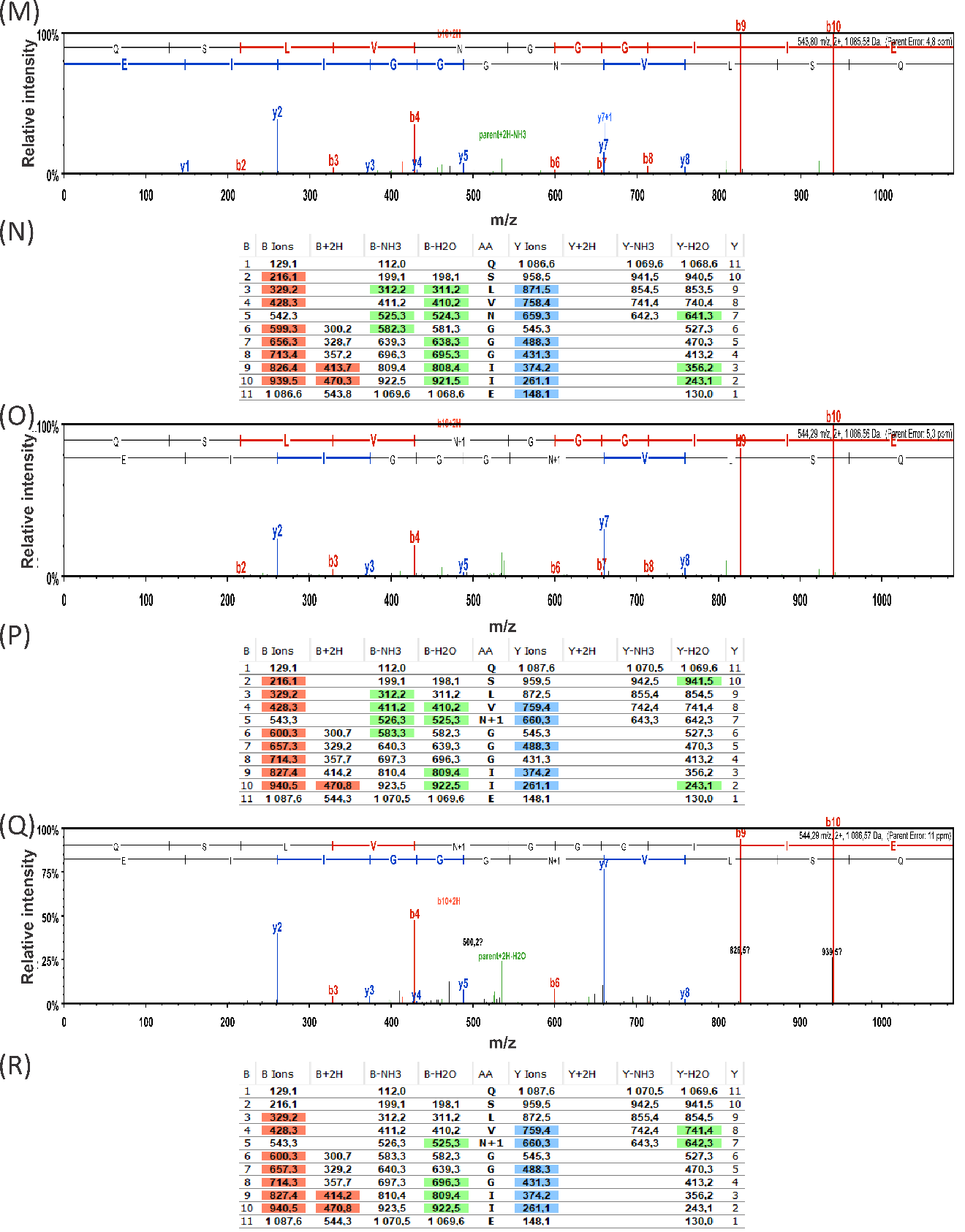
**

**Supplemental Figure 3 (continued)**

**
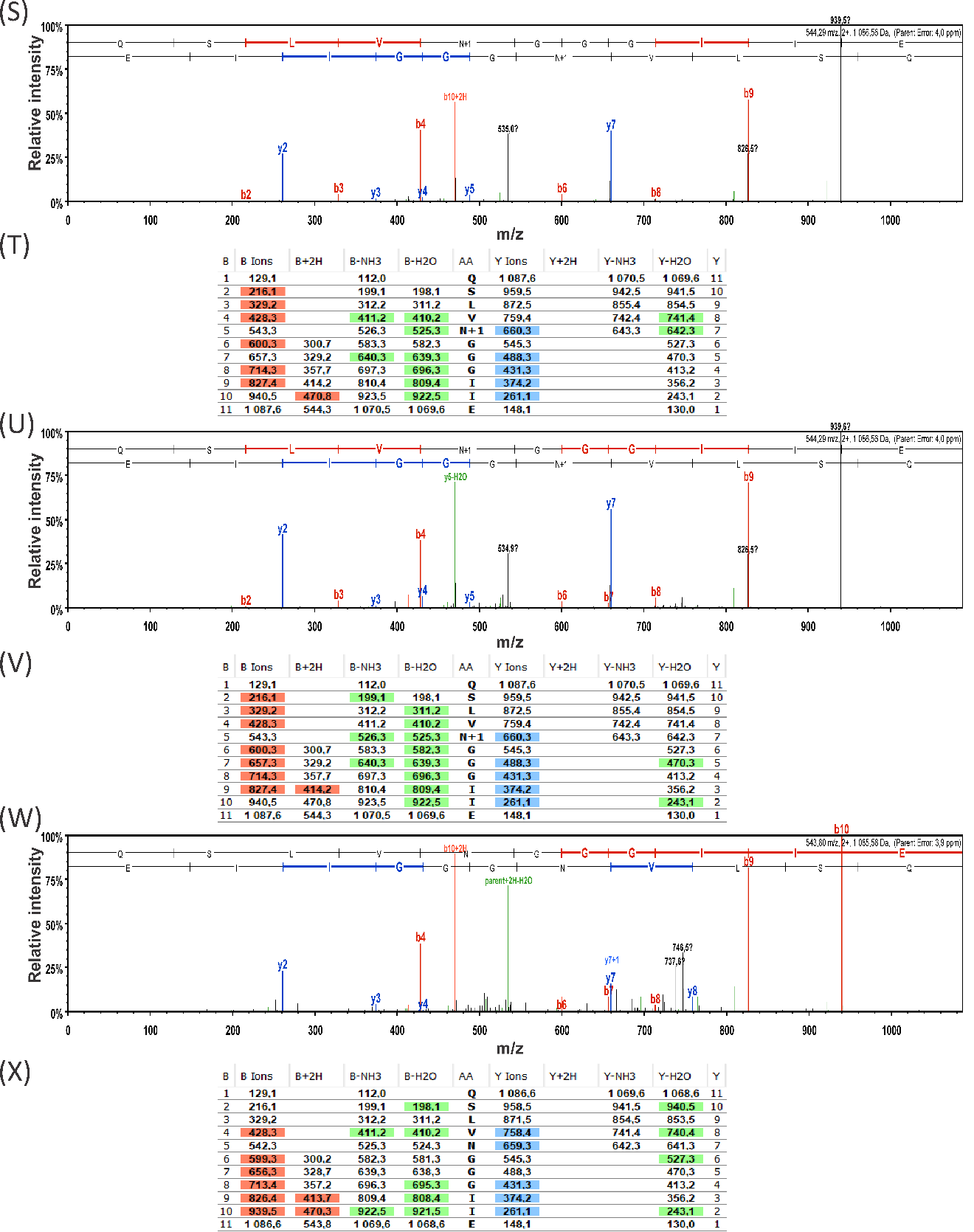
**

**Supplemental Figure 3. MS/MS sequence data of all peptides carrying Ser^600^ obtained after tryptic digestion and LC MS/MS analysis of purified recombinant LOX2^WT^.** The fragmentation spectra of the peptides are shown in panels A, C, E, G, I, K, M, O, Q, S, U and W. For each spectrum, the ion table displaying the predicted B and Y series for the peptide is shown just below in panels B, D, F, H, J, L, N, P, R, T, V and X respectively. The peptide sequences are shown at the top of the spectrum panels and in the table columns marked AA. Matches between peaks detected in the spectrum and the expected theoretical mass in the table are marked by color highlighting. Note that results shown in panels A-F result from an incomplete tryptic digestion (one missed site). Data shown in panels K, L, O, P, Q, R, S, T, U and V result from Asn-deaminated peptides. No peptide carrying a phosphor-Ser^600^ was recovered in the analyses. Data were analyzed by the Scaffold 5.1.0 software.


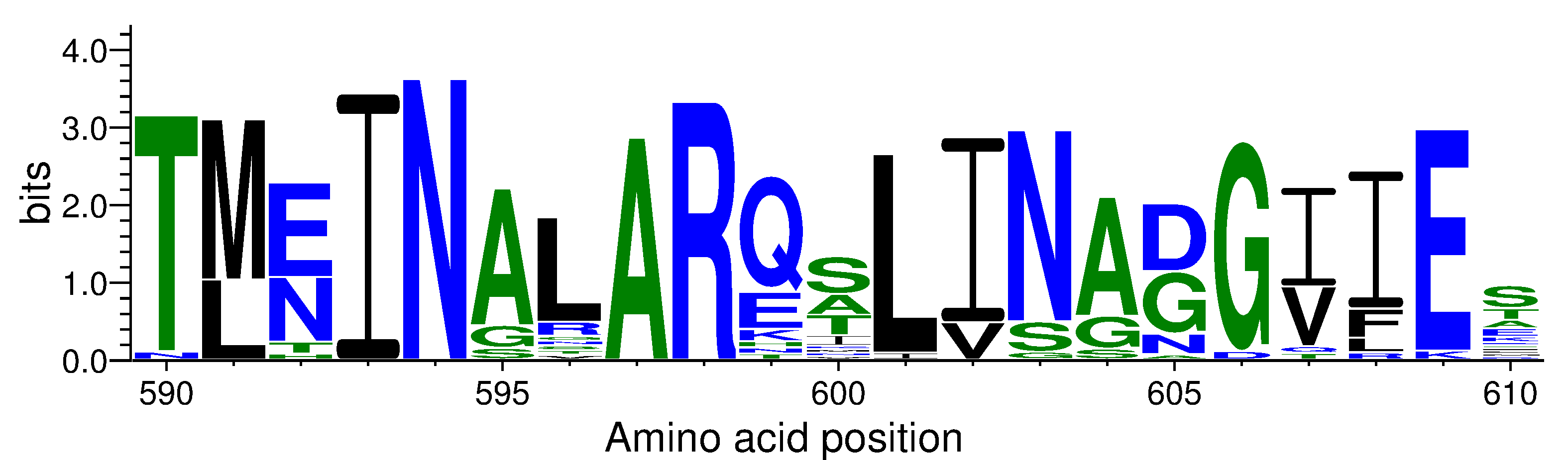


**Supplemental Figure 4**. **Amino acid conservation at and around the Ser identified as being a potential phosphosite in plant LOXs involved in defense**. LOX involved in plant defense are listed in Supplemental Table 1. Amino acid position refers to the arabidopsis LOX2 sequence numbering with a phosphoSer at position 600. The representation was done using Weblogo 3 (<http://weblogo.threeplusone.com/> ([Crooks GE](http://threeplusone.com/), [Hon G](http://compbio.berkeley.edu/), [Chandonia JM](http://compbio.berkeley.edu/), [Brenner SE](http://compbio.berkeley.edu/people/brenner/) (2004) WebLogo: A sequence logo generator, *Genome Research*, 14:1188-1190)).
